# Pandemic Preparedness of Care Homes Serving Older Adults in the Wake of COVID-19: An Exploratory Study

**DOI:** 10.1093/geroni/igaa057.1558

**Published:** 2020-12-16

**Authors:** Iftekhar Amin

**Affiliations:** University of North Texas at Dallas, Dallas, Texas, United States

## Abstract

Recent COVID-19 pandemic has disproportionately affected the older adult population worldwide. According to CDC, among older adults over 60 years the risk increases with age, with the highest risk of serious illness and death among those over 80 years. While public has been receiving messages about the risks and how to take preventive measures, it is not clear how the care homes serving older adults have been preparing. Data have been collected as part of an ongoing study from 30 independent living, assisted living, and memory care facilities across the United States. The centers were selected with a snowball sampling technique. Administrators of the centers were interviewed with a semi-structured questionnaire. It was apparent that although awareness of risks was high, preparation appears to be inadequate with little resources available at the time of the survey. Variation of preparedness based on the sociodemographic characteristics of the residents suggests that homes that serve predominantly minority and economically disadvantaged have greater likelihood of lacking preventive resources. It is critical that facilities serving older adults be prepared to ensure an effective healthcare response in the wake of novel viruses, such as COVID-19.

